# High Plasma Fibrinogen is Correlated With Poor Response to Trastuzumab Treatment in HER2 Positive Breast Cancer

**DOI:** 10.1097/MD.0000000000000481

**Published:** 2015-02-06

**Authors:** Yu-Lin Liu, Qing Lu, Ji-Wei Liang, Yu Xia, Wei Zhang, Bao-Quan Hu, Fang-Fang Shang, You-Ran Ji, Jun Wang, Qiang Wang, Bing Liang

**Affiliations:** From the Department of Clinical Laboratory, 401st Hospital of People's Liberation Army, Qingdao 266071, China (Y-LL, J-WL, YX, JW, BL); Department of Pathogenic Biology, Qingdao University, Qingdao 266000, China (Y-LL, BL); Medical Affairs Department, 401st Hospital of People's Liberation Army, Qingdao 266071, China (QL, Y-RJ); Department of Pathology, the 401st hospital of People's Liberation Army, Qingdao 266071, China (WZ, F-FS, QW); Department of Breast Diseases, Southwest Cancer Center, Southwest Hospital, Third Military Medical University, Chongqing 400038, China (B-QH); Institute of Pathology and Southwest Cancer Center, Southwest Hospital, Third Military Medical University, Chongqing 400038, China (QW).

## Abstract

Some of HER-2 positive breast cancer patients failed to trastuzumab treatment. Recent reports have indicated the correlation between plasma coagulation parameters and clinical characteristics in breast cancer. The aim of this study was to analyze the role of coagulation parameters in trastuzumab treated patients.

Coagulation parameters from trastuzumab treated breast cancer patients were retrospectively studied from 2006 to 2010. The correlation between routine coagulation levels and clinical characteristics were analyzed, including prothrombin time (PT), activated partial thromboplastin time (APTT), thrombin time (TT), fibrinogen (Fib) and D-dimer (DD). The Kaplan–Meier analysis and Cox regression hazard model were applied to assess their effect on prognosis.

Totally 102 hospitalized breast cancer patients who received trastuzumab were collected and followed-up. All patients were HER-2 positive advanced breast cancer, with a median age of 45 years old. Extended PT, APTT, and TT were found in trastuzumab treatment non-effective group, as well as increased Fib and DD. But significant increase was only found in Fib. High Fib status (Fib > 2.88 g/L) was correlated with clinical characteristics, such as pathological grade, and reversely correlated with PTEN expression. More importantly, poor disease-free survival (DFS) and overall survival (OS) to trastuzumab treatment were found in high Fib breast cancer patients.

This retrospective study suggests high Fib status was correlated with poor treatment response to trastuzumab. Our findings indicated that Fib > 2.88 should alert physicians to consider a pretreatment for reducing Fib levels before trastuzumab treatment in HER-2 positive breast cancer patients.

## Abstract 2

Supplemental Digital Content is available in the text

## INTRODUCTION

Therapeutic monoclonal antibody promoted the progression of clinical treatment for some advanced malignancies,^1^ especially in conjunction with chemotherapy or radiotherapy.^2,3^ Monoclonal antibody targeted on tumor antigen for the treatment efficiency, and caused only a few adverse effects, including tumor lysis syndrome.^4,5^ Trastuzumab had been widely used in clinical treatment for breast cancer in last decade. However, remarkable differences in clinical response were found in trastuzumab-treated patients.^6,7^ Some patients included in the therapeutic range did not benefit from the monoclonal antibody targeted therapy. Currently, the mechanism studies of monoclonal antibody treatment failure were focused on tumor cells and immune cells.^4^ It is still not yet clear about the role of internal environmental homeostasis, especially coagulation related indicators, in this procession.

Therapeutic monoclonal antibodies were injected into the blood, and reached tumor tissue and conferred tumor-killing effect. The transportation process will be affected inevitably by plasma microenvironment. Moreover, hypercoagulable status of plasma was usually found in the breast cancer patients.^8–11^ However, the correlation between elevated coagulation parameters and treatment response in breast cancer patients are still disputing.

Here, a retrospective study was performed to follow-up breast cancer patients who received trastuzumab therapy and collected their coagulation test results before treatment. The correlation between coagulation parameters and treatment response to trastuzumab were compared and analyzed.

## MATERIALS AND METHODS

### Patients and Plasma

The retrospective study included breast cancer patients who received trastuzumab treatment in 401st Hospital of People's Liberation Army and Southwest Hospital from 2006 to 2010. Another 34 breast cancer patients with high fibrinogen (Fib) in the same periods were studied as control. The criteria of cases inclusion were: (1) newly diagnosed breast cancer which pathologically confirmed; (2) complete results of coagulation tests, including prothrombin time (PT), activated partial thromboplastin time (APTT), thrombin time (TT), Fib and D-dimer (DD); (3) complete clinical and pathological data, including age, histological type, tumor size, lymph nodes metastasis, TNM stage, expression of ER, PR, and HER-2. Exclusion criteria were: (1) patients with previous thrombosis history; (2) serious infection or trauma disease history within nearly a month; (3) previous arrhythmia history; (4) using anticoagulant or hemostatic drugs. Totally 102 female cases were included, which were all HER2-positive advanced breast cancer patients. Informed consent was approved for each patient by the ethics committee of 401st Hospital and Southwest Hospital complying with the research proposals, including written informed consent and availability of plasma, and follow-up data. Clinical information of each specimen was obtained by the medical records, telephone or written correspondence, and death certificate.

### Plasma Coagulation Test

Venous blood samples of 5 mL for plasma coagulation test were collected in tubes with sodium citrate in the morning before trastuzumab treatment. The plasma coagulation parameters, including PT, APTT, TT, Fib, DD, were tested with a SYSMEXCA7000 automatic coagulation analyzer (Sysmex Corporation, Kobe, Japan) using latex-enhanced immunoturbidimetric assay in both Laboratories. Commercial standard reagents were used as control. Normal reference ranges were: PT 9.8–13.7 seconds, APTT 21.4–32.7 seconds, TT 14–21 seconds, Fib 1.8–3.7 g/L, DD 0–392 ng/mL.

### Immunohistochemical Staining for PTEN

Formaldehyde fixed tumor specimens were embedded in paraffin for histological sections, which deparaffinized in xylene, dehydrated with graded alcohol solution. Immunohistochemical staining for Phosphatase and tensin homolog (PTEN) was performed with a rabbit monoclonal antibody (Abcam, Cambridge, UK). The sections was incubated in citrate buffer pH 6.0 for antigen retrieval, then incubated with the primary antibody at 1:200 dilution overnight at 4 °C. Next, they were incubated with the horseradish peroxidase-conjugated secondary antibody after rinsed with phosphate buffer solution (PBS), followed by incubation with diaminobenzidine staining, and counterstaining with hematoxylin blue. Control IgG was used as negative control. Two pathologists, who were blinded to the pathological diagnosis and clinical data, observed the immunohistochemical staining results.

### Statistical Analysis

All data were analyzed with SPSS 19.0 software. Correlations between clinical response and clinical characteristics were analyzed by Chi-square test or Spearman's rank correlation (*r*) analysis, as well as Fib status and categorical variables. Normal distribution measurement data (PT, APTT, Fib, TT) were shown with *x* ± *s*, using *t* test or ANOVA analysis. Comparison between the two groups were performed with SNK test; data of skewed distribution (DD) was shown with median and interquartile range represents [M (QR)], using the Wilcoxon test. A receiver operating characteristics (ROC) analysis was performed on measured Fib levels according different response. The optimal PMR cutoff value was identified according to the Youden index. Survival curves were constructed using the Kaplan–Meier method, and survival differences were evaluated by the log-rank test. Univariate and multivariate analysis were done with a Cox proportional hazards model to determine the effect of Fib status, and other clinical variables (age, size, histological grade, lymph node status, and ER/PR expression) on DFS and OS. Hazard ratios and their corresponding 95% confidence intervals were provided with quantitative information for the relevance of the statistical analysis. Differences with *P* value of 0.05 or less were considered to be statistically significant.

## RESULTS

### Clinical Characteristic Comparison Between Trastuzumab Treated Effective Group and Non-Effective Group

Totally 102 advanced breast cancer patients were included into the study who received trastuzumab treatment. All the cases were female, with a median age of 45 years old. Clinical criteria were compared between effective group and non-effective group (*n* = 74 vs 24, Table 1). Criteria for treatment non-effective response were based on recurrence, metastasis or death occurred within 5 years after trastuzumab treatment. However, no significant differences were found in age (*P* = 0.753), tumor size (*P* = 0.675), pathological grade (*P* = 0.061), lymph node metastasis (*P* = 0.077) and the expression of ER (*P* = 0.930), or PR (*P* = 0.736).

**TABLE 1:**
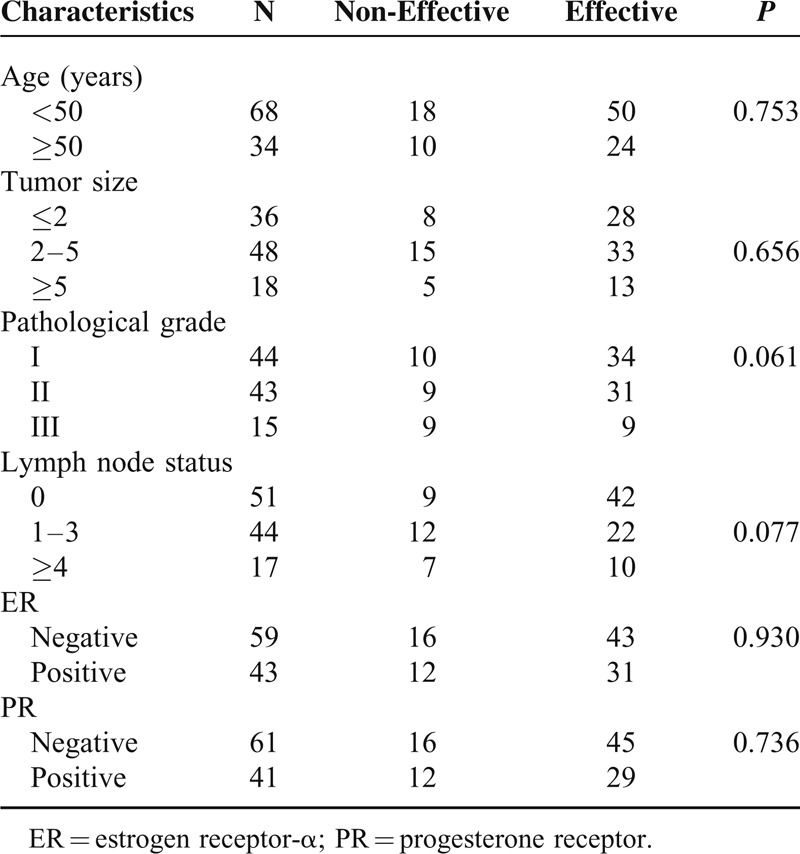
Clinical Characteristics of All 102 Trastuzumab Treated Breast Cancer Patients

### Levels Comparison of Coagulation Factors Between Effective Group and Non-Effective Group

The plasma coagulation parameters were compared between effective group and non-effective group (Table 2). Fib levels were significantly increased in non-effective patients, compared with effective breast cancer patients [(3.27 ± 0.73) g/L vs (2.75 ± 0.69) g/L, *P* < 0.001]. However, non-effective patients showed only slightly extended PT [(11.21 ± 0.62) s vs (10.90 ± 0.66) s, *P* = 0.435], APTT [(26.89 ± 3.55) s vs (25.38 ± 3.58) s, *P* = 0.193] and TT [(16.88 ± 1.27) s vs (16.40 ± 1.16) s, *P* = 0.447)], and slightly increased DD [152 (154) ng/mL vs 149 (194) ng/mL, *P* = 0.261] levels. The results showed that compared with the effective group, Fib was found with significant increase in non-effective group (*P* < 0.001), but no significant increase was observed in PT, APTT, TT, and DD (Table 2).

**TABLE 2:**
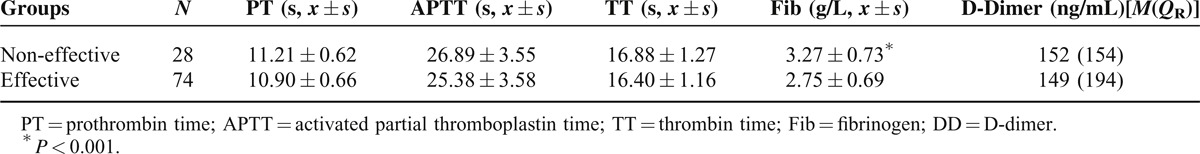
Coagulation Parameters Comparison Between Effective Group and Non-Effective Group

### Critical Analysis for Fib Level for Predicting Response for Trastuzumab Therapy

**FIGURE 1:**
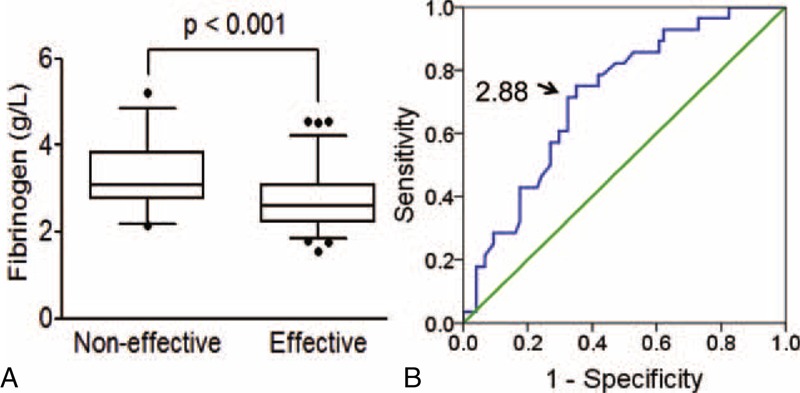
Critical analysis for Fib level for predicting response for trastuzumab therapy. A. Higher level of Fib was found in non-effective group for trastuzumab therapy than effective group (*P* < 0.002); B. Receiver Operating Characteristics (ROC) analysis was performed on measured Fib levels according different response in patients (*n* = 102). The optimal Fib level cutoff value was identified according to the Youden index at 2.88, that is, the point on the curve farthest from chance.

Significantly higher level of Fib was found in non-effective group for trastuzumab therapy than effective group (*P* < 0.001, Figure 1A). A ROC analysis was performed for Fib level cutoff point to prognosis the efficiency of trastuzumab treatment (Figure 1B). The area under the ROC-curve was calculated to 0.72 (95% CI: 0.61–0.82), indicating that the probability of observing a higher Fib level in the non-effective patients was about 71%. Fib levels above 2.88 were considered as high Fib group, whereas Fib levels below 2.88 were classified low Fib group. And high Fib status was correlated with poor response to trastuzumab treatment.

### Relationship Between Fib Status and Clinical Features

Table 3 listed the relationships between Fib status and clinical features in our study. Fib status was statistically associated with histological grade. Higher Fib levels were found in patients with higher histological grade than those with lower grade (*P* < 0.05). However, we failed to detect relationships between Fib status and other clinical characteristics, including age (*P* = 0.110), tumor size (*P* = 0.652), lymph node status (*P* = 0.477) and ER (*P* = 0.098) or PR (*P* = 0.780) expression.

**TABLE 3:**
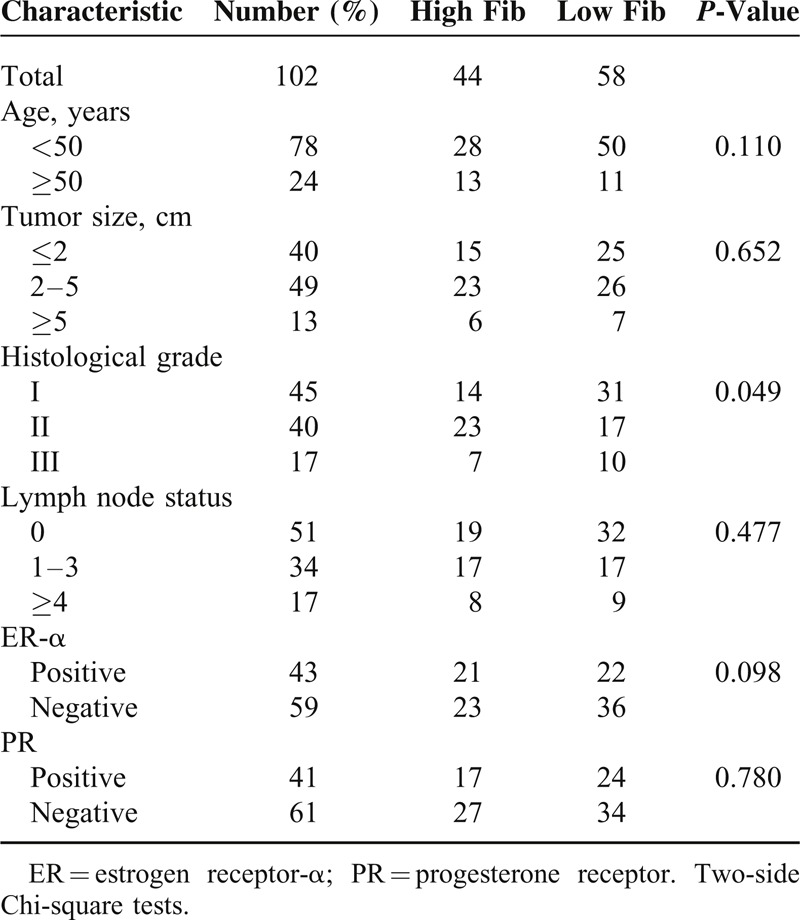
Correlations of Fibrinogen Status and Clinical Characteristics

### Correlation of Fib Status With PTEN Expression

**FIGURE 2:**
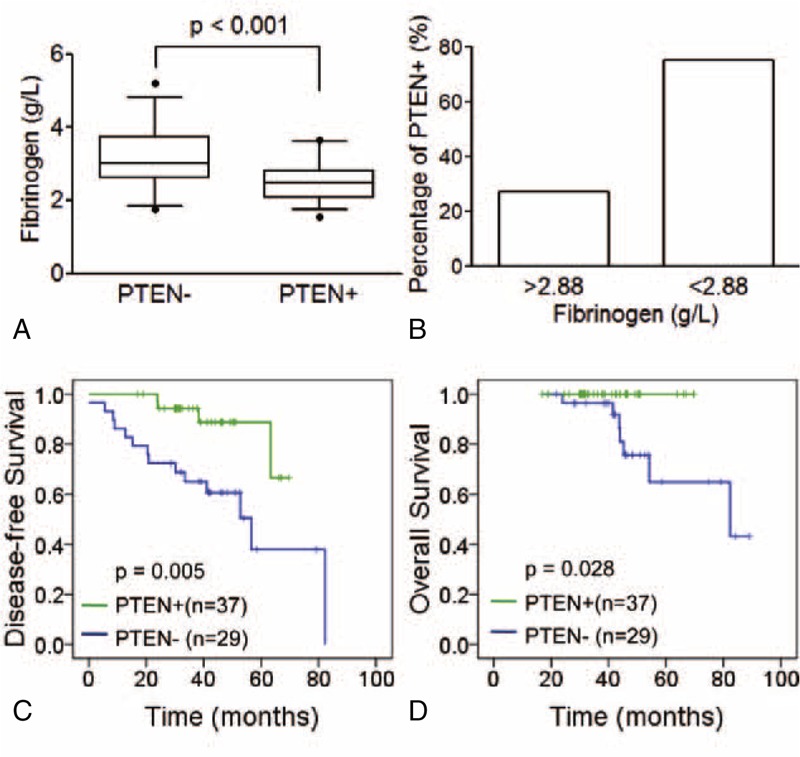
Correlation of Fib levels with PTEN. A. Higher Fib levels were found in PTEN positive group (*P* < 0.001). B. Higher percentage of PTEN positive patients were found in high-Fib groups (26.9% vs 75.0%). Low PTEN expression was associated with poor prognosis of patients: disease-free survival (C, *P* = 0.005) and overall survival (D, *P* = 0.028).

PTEN modulated trastuzumab sensitivity in HER2-overexpressing breast cancer, because of its opposition to phosphoinositide 3-kinase (PI3K) downstream signaling.^12^ Based on the correlation of high Fib status and trastuzumab treatment failure as shown above, we further evaluated the relationship between Fib status and PTEN. We found PTEN expression was reversely correlated with Fib levels (*P* < 0.001, Figure 2A). However, no correlation between histological grade and PTEN expression was found (*P* = 0.115, supplemental data http://links.lww.com/MD/A180). More patients were found positive for PTEN in low Fib group (Figure 2B). Consistent with previous reports, shorter disease-free survival (DFS) was observed in low PTEN expression groups than high ones (*P* = 0.005, Figure 2C), as well as significant difference in overall survival (OS) in our study (*P* = 0.028, Figure 2D).

### Effect of Fib Status on Patient Prognosis

**FIGURE 3:**
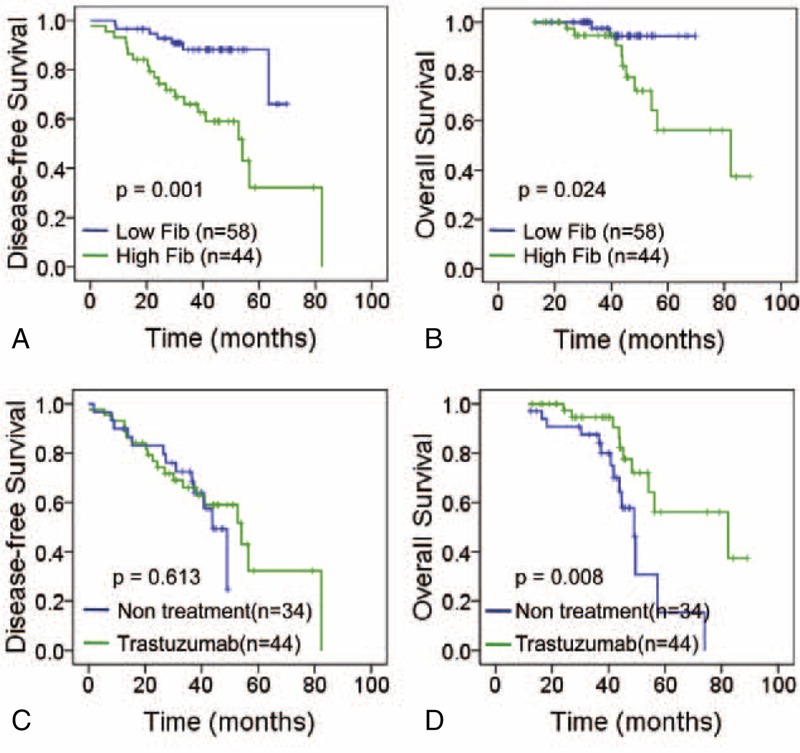
Effect of Fib statuses on patient prognosis. Kaplan–Meier analysis of Fib status was performed on disease-free survival (A, *P* = 0.001) and overall survival (B, *P* = 0.024) of trastuzumab therapy in breast cancer patients. Kaplan–Meier analysis of breast cancer patients of high Fib status when treated with trastuzumab therapy or not: disease-free survival (C, *P* = 0.613) and overall survival (D, *P* = 0.008).

Follow-up data was available for all patients with a median follow-up time of 41.2 months (range: 13.0–89.1); during the follow-up period, 12 patients (11.8%) had died from recurrence or distant metastasis. Prognosis were analyzed with Kaplan–Meier curves to show that high Fib status was statistically associated with worse DFS and OS in trastuzumab treated patients (*P* = 0.001, *P* = 0.024, Figure 3A and B).

Furthermore, a Cox multivariable proportional hazard model was constructed to examine independent prognostic significance of high Fib status. Other potential factors (age, size, grade, lymph node metastasis, and ER/PR expression) were all took into account. Among them, high Fib status was significantly correlated with worse prognosis in both DFS and OS (*P* = 0.002, *P* = 0.003, Table 4). Moreover, the results of Cox univariate analysis confirmed that high Fib status was a significant predictor for shorter DFS and OS in trastuzumab treatment, independent of other factors (*P* = 0.002, *P* = 0.004. Table 5). Moreover, although no significant improvement of DFS was observed in the patients with high Fib treated with trastuzumab compared with no-treatment (*P* = 0.613, Figure 3C), significant improvement was observed in OS in trastuzumab treated group (*P* = 0.008, Figure 3D).

**TABLE 4:**
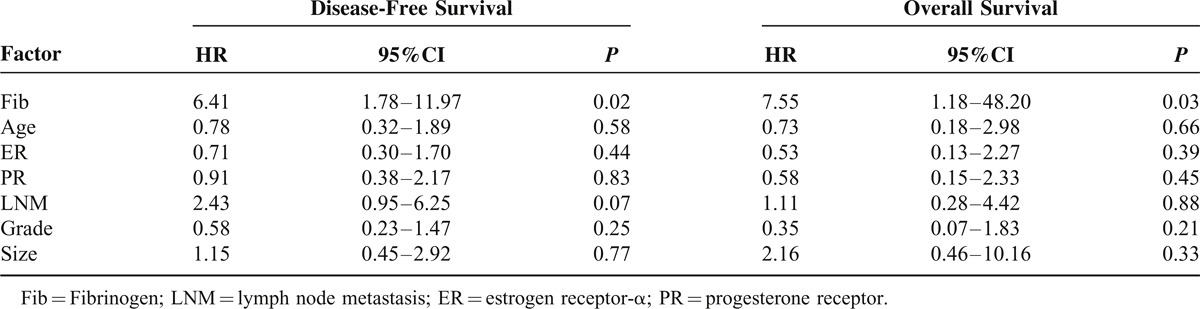
Multivariate Analyses of Disease-Free Survival and Overall Survival

**TABLE 5:**
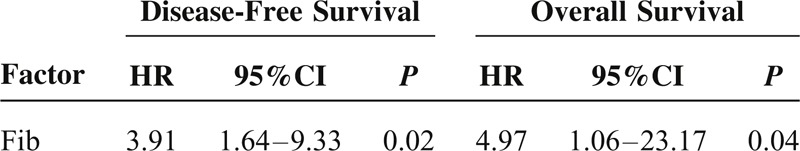
Univariate Analyses of Disease-Free Survival and Overall Survival

## DISCUSSION

Our study here followed up the trastuzumab treatment response of breast cancer patients, collected coagulation parameters tests including PT, APTT, TT, Fib, and DD, and clarified the difference in coagulation parameters between effective group and non-effective group. Higher levels of plasma Fib were significantly correlated with clinical characteristics such as pathological grade, lymph node metastasis and ER expression. Most notably, high Fib status showed a significant predicting value for poor clinical response to trastuzumab treatment.

Plasma Fib level is an important indicator reflecting the hypercoagulable status in blood. Several clinical studies had reported that the majority of breast cancer patients showed hypercoagulable state.^10,13,14^ High levels of coagulation parameters were correlated with a variety of clinical and pathological factors in a series of carcinoma or sarcoma, such as breast cancer,^15^ oesophageal cancer,^16^ colorectal cancer,^17,18^ ovarian cancer,^19^ advanced non-small-cell lung cancer,^20^ gastric cancer.^21^ High Fib status played an important role in development and progression of tumors,^11^ such as providing microenvironment for micrometastases, survival and proliferation of tumor cells, protecting tumor cells from immune cells attack,^22,23^ which were all closely associated with poor prognosis of carcinoma. However, previous studies remain controversial on these correlations.^19,24^ Distinguished hypercoagulable state was observed in breast cancer patients, thus the evaluation of plasma coagulation parameters cannot rely solely on normal criteria. Our results here revealed that a significant elevation of coagulation parameters (Fib) in breast cancer patients was correlated with failure in trastuzumab treatment. It is reasonable to determinate a criteria for cutoff value of Fib level based on clinical response to treatment, which will contribute to analyze the correlation between Fib levels and clinicopathological factors.

Trastuzumab had been widely used in clinical trials, which showed an improved therapeutic effect in clinical treatment of breast cancer patients.^7,25^ Anti-tumor activity of Trastuzumab was mainly based on two ways: Firstly, to combine with targeted proteins (HER-2) of tumor cell, inhibiting cell signaling transduction in the cancer cells^26,27^; second, to activate immune system, regulating immune mechanism to kill tumor cells.^28,29^ Fewer adverse reactions were found in trastuzumab immunotherapy, the effect of combined chemotherapy or radiotherapy was better in clinical treatment. However, some patients failed with clinical response to trastuzumab and suffered poor prognosis.^6,7^ Previous studies for the mechanism of poor clinical response are summarized in two aspects: first, trastuzumab failed to block the signal pathway, which may be due to the low expression or mutation of targeted antigen in the tumor cells themselves, resulted in low binding of antibodies in tumor cells^12,30^; or other signaling pathway activated by related proteins which expressed in the tumor cells; Second, weakened immune system activation, which due to the specific isotypes^31,32^ and insufficient number of monoclonal antibody^33^ to effectively activate the immune response, or reduced Fcγ receptor expression in NK cells,^34,35^ or eliminated or dysfunctioned immune cells.^4,36^ Our study found that trastuzumab treatment failure in breast cancer patients was significantly correlated with high Fib status, indicating that hypercoagulable states may directly affect trastuzumab treatment efficiency. Trastuzumab therapy significantly improved the prognosis of high Fib patients, especially overall survival. However, high Fib status reduced the response to trastuzumab therapy. Our study suggests that reducing blood hypercoagulable levels may improve patient's response to trastuzumab treatment, especially Fib levels. This assumption still needs prospective clinical trials to be verified.

PI3K and downstream signaling inhibition is the main target for trastuzumab treatment. Previous studies have revealed that PTEN opposite to PI3K signaling to modulate trastuzumab sensitivity in HER2-overexpressing breast cancer. Our study here observed a reverse correlation between Fib levels and PTEN expression, which may shed a light to the mechanism of PTEN regulation in vivo. However, whether PI3K signaling was included in high Fib status induced trastuzumab treatment failure was still needed to further study.

In conclusion, our study indicated that hypercoagulable state in breast cancer patients, especially high Fib status, was an adverse factor for trastuzumab therapy. More importantly, it provided proofs to pretreat patients before targeted therapy to improve therapeutic effect. Coagulation parameters tests of malignant patients were an additional data for disease diagnosis and prognosis.^37^ They also had important reference value for clinical treatment choice, especially trastuzumab therapy.
